# Isolated Gallbladder Melanoma: A Diagnostic Conundrum of Primary vs. Metastatic Disease

**DOI:** 10.7759/cureus.77428

**Published:** 2025-01-14

**Authors:** Ryan Patterson, Jessica Wernberg, Rohit Sharma

**Affiliations:** 1 General Surgery, Marshfield Medical Center, Marshfield, USA; 2 Surgical Oncology, Marshfield Medical Center, Marshfield, USA

**Keywords:** gallbladder, immunotherapy, melanoma, metastatic, primary

## Abstract

Melanoma is a malignant proliferation of melanocytes originating from neural crest cells. Gallbladder melanoma is typically reported as a metastatic disease involving the adjacent liver. Thus, isolated gallbladder melanoma must be either a primary tumor originating within the gallbladder without evidence of metastasis or an isolated metastatic lesion from an unknown or regressed primary site. Regardless of its classification as primary or metastatic, isolated gallbladder melanoma is an extremely rare diagnosis. We present a case of incidentally discovered isolated gallbladder melanoma in a resected specimen from a patient undergoing surgery for symptomatic cholelithiasis. An exhaustive workup failed to identify a primary site outside the gallbladder. The case was reviewed at a multidisciplinary tumor board, and the patient was started on immunotherapy. This case report aims to highlight this rare entity, discuss potential management strategies, and contribute to the limited body of literature on the subject.

## Introduction

Melanocytes are typically found in ectodermal derivatives such as the skin, eyes, and mucosa, but are not commonly present in endodermal derivatives like the gallbladder. While primary melanoma of the gallbladder is a debated clinical entity, it remains theoretically possible. However, gallbladder melanoma is most often identified as part of a metastatic disease pattern. Autopsy studies of patients with metastatic melanoma have reported biliary metastases in 4-20% of cases, typically in the context of widespread peritoneal disease [1]. Additionally, no identifiable primary lesion is found in 4-12% of metastatic melanoma cases [2].

Here, we present a case of incidental gallbladder melanoma, detailing the diagnostic workup, treatment options, and a brief review of the literature.

## Case presentation

**Figure 1:**
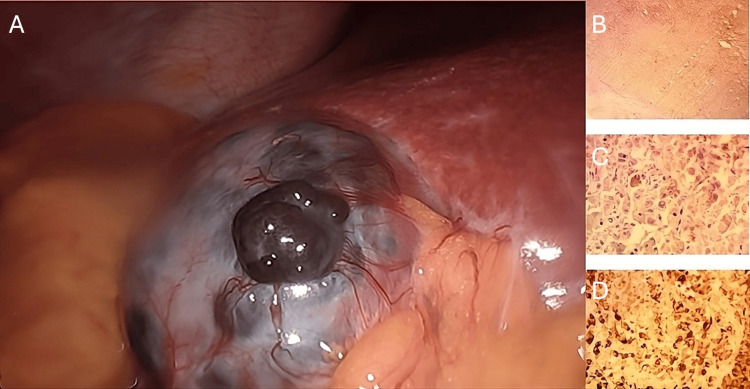
Incidental lesions observed on the gallbladder surface during laparoscopy (A) Exophytic, darkly pigmented lesion identified during laparoscopic cholecystectomy. (B) Low-power view showing melanoma involvement of the gallbladder wall. (C) High-power view of melanoma cells with prominent nucleoli and dusky brown, coarsely pigmented cytoplasm. (D) Positive immunohistochemical staining for melanoma cocktail (courtesy of Dr. Ryan Bemke).

A 62-year-old Caucasian female presented with one day of right upper quadrant (RUQ) pain. Her medical history included morbid obesity, and her father had a history of non-melanoma skin cancer. She was a former smoker. Physical and laboratory examinations were unremarkable except for tenderness to palpation in the RUQ. A RUQ ultrasound revealed a large 5 cm stone and sludge. She was diagnosed with symptomatic cholelithiasis and, one month later, underwent elective laparoscopic cholecystectomy. Intraoperatively, an exophytic, darkly pigmented lesion approximately 2 cm in size (Figure 1) was identified on the gallbladder, with no lesions observed on the visualized hepatic surface. The gallbladder was aspirated, but otherwise not entered, and was removed in a specimen retrieval bag. No irrigation was performed during the operation.

Histology revealed nests of melanoma extending transmurally from the mucosa, an equivocal cystic duct margin, and a positive cystic lymph node (LN; one of two). Stains were S100/melanin positive and CD68 negative. Molecular markers identified the lesion as NRAS and TERT mutated, while BRAF, KIT, GNA11/GNAQ, and BAP1 were wild type. Investigations for an alternate primary site, including PET-CT, esophagogastroduodenoscopy (EGD)/colonoscopy, and dermatological and ophthalmological exams, were negative. The case was presented at the multidisciplinary tumor board (MDTB), and treatment with pembrolizumab (Keytruda, Merck, Rahway, New Jersey, United States) was recommended. Restaging scans five months later showed FDG-avid aortocaval LNs and soft tissue adjacent to cystic duct clips. Ipilimumab (Yervoy, Bristol-Myers Squibb, New York, New York, United States) was added for four cycles. Tumor-infiltrating lymphocyte (TIL) therapy was not offered, as the patient presented prior to FDA approval for TIL therapy for patients with progression of melanoma while on first-line therapy. Post-ipilimumab PET scans showed improvement in aortocaval LNs and a left lower lung lobe avid density. Chest CT revealed interval enlargement of the pulmonary nodule, and biopsy showed chronic organizing pneumonia. Immunotherapy was discontinued due to hypoxia, requiring supplemental oxygen and steroids. A CT scan one month later showed metastatic deposits in the greater omentum, duodenum, and stomach. She is currently alive, three years post-cholecystectomy, but unfortunately has developed brain metastases unresponsive to radiation therapy. She is currently maintained on temozolomide (Temodar, Merck).

## Discussion

In the English literature, only nine cases have been reported of either primary gallbladder melanoma without metastatic disease or isolated metastatic melanoma of the gallbladder without an identifiable primary lesion (Table 1) [3-7].

**Table 1:** Review of isolated gallbladder melanoma in the English literature A review of the literature highlights the rarity of isolated gallbladder melanoma, whether primary or metastatic [3-7]. Heath and Womack documented six primary cases, three of which were isolated to the gallbladder, and 13 metastatic cases, two of which were isolated to the gallbladder. Giannini et al. reported four cases of isolated gallbladder melanoma, all classified as metastatic.

Clinical study	Primary or metastatic	Number of cases	Isolated gallbladder melanoma
Heath and Womack (1988) [3]	Primary	19	5 cases
Giannini et al. (2016) [4]	Metastatic	38	4 cases
Jeon et al. (2021) [5]	Metastatic	4	0 cases
Furumoto et al. (2013) [6]	Metastatic	1	0 cases
Cassou-Mounat et al. (2019) [7]	Metastatic	1	0 cases

Heath and Womack (1988) proposed criteria for primary gallbladder melanoma, which include a single focus arising from the mucosa with junctional activity and the absence of other primary lesions following a thorough workup. Junctional activity is the most reliable histological indicator of a primary melanoma; however, it may be absent due to rapid tumor growth in true primary cases. It has also been observed in cases of metastatic disease [3]. The presented case exhibits features associated with both primary and metastatic melanoma, as summarized in Table 2.

**Table 2:** Characteristics of primary vs. metastatic lesions of melanoma ^*^ The far-right column demonstrates that our patient exhibited characteristics of both primary and metastatic disease.

Characteristic	Primary	Metastatic	Our patient
Mucosal involvement	Involved	Not involved	Involved
Junctional activity^*^	Present	Absent	Absent
Focality	Single	Multiple	Multiple
Precursor lesion	Present	Absent	Absent
Alternate primary workup	Negative	Present	Negative

No further surgical intervention was offered to our patient due to the risk of widespread peritoneal disease associated with gallbladder entry and aspiration, as well as a positive cystic node and features more consistent with metastatic disease, despite some mixed results. Patients with preoperatively diagnosed isolated gallbladder melanoma have a median survival of 39 months, compared to 10 months for those with widespread disease. Additionally, patients who undergo cholecystectomy have a median survival of 19 months, compared to six months for those treated nonoperatively [8]. There is insufficient evidence to recommend an open or laparoscopic approach as long as the gallbladder is not entered and a specimen retrieval bag is used. Katz et al. reported three cases with a laparoscopic approach, two of which had port site recurrence; however, both patients had other sites of disease at the time of surgery. Subsequent cases of laparoscopic cholecystectomy, including our own, have been reported without port site recurrence [9].

## Conclusions

Isolated gallbladder melanoma is an extremely rare diagnosis. When identified, the patient should undergo a thorough workup, including an ocular exam, flexible nasopharyngoscopy, EGD/colonoscopy, pelvic exam, dermatological exam, and PET-CT. The specimen should be reviewed by a specialized GI histopathologist, focusing on mucosal involvement and junctional activity, and testing should be conducted for available molecular targets to guide immunotherapy. The case should also be presented to the MDTB. Further surgical intervention should depend on the staging workup, intraoperative gallbladder entry, and the availability of systemic therapy. Most documented cases of isolated gallbladder melanoma occurred before the shift in systemic treatment from chemotherapy to immunotherapy. Future management will likely focus more on actionable aberrant molecular alterations than on the site of origin. In cases of preoperatively identified isolated gallbladder melanoma, surgery should be offered, as it improves both disease-free and overall survival. The choice between minimally invasive or open surgery should be at the discretion of the surgeon. The aim of this case report is to contribute to the literature in this evolving field.
